# Interventions to Reduce Harm from Smoking with Families in Infancy and Early Childhood: A Systematic Review

**DOI:** 10.3390/ijerph120303091

**Published:** 2015-03-16

**Authors:** Nicola Brown, Tim Luckett, Patricia M. Davidson, Michelle Di Giacomo

**Affiliations:** 1 Centre for Cardiovascular and Chronic Care, Faculty of Health, University of Technology, P.O. Box 123, Broadway, New South Wales 2007, Sydney Australia; E-Mails: Tim.Luckett@uts.edu.au (T.L.); pdavidson@jhu.edu (P.M.D.); Michelle.DiGiacomo@uts.edu.au (M.D.G.); 2 John Hopkins University, School of Nursing, Baltimore, MD 21205, USA

**Keywords:** child, family, smoking, smoking cessation, second hand smoke, antismoking socialisation

## Abstract

Exposure to adult smoking can have deleterious effects on children. Interventions that assist families with smoking cessation/reduction and environmental tobacco smoke (ETS) avoidance can improve child health outcomes and reduce the risk of smoking initiation. The purpose of this review was to describe the state of the science of interventions with families to promote smoke-free home environments for infants and young children, including parent smoking reduction and cessation interventions, ETS reduction, and anti-smoking socialisation interventions, using the socio-ecological framework as a guide. A systematic review of peer-reviewed articles identified from journal databases from 2000 to 2014 was undertaken. Of 921 articles identified, 28 were included in the review. Considerable heterogeneity characterised target populations, intervention types, complexity and intensity, precluding meta-analysis. Few studies used socio-ecological approaches, such as family theories or concepts. Studies in early parenthood (child age newborn to one year) tended to focus on parent smoking cessation, where studies of families with children aged 1–5 years were more likely to target household SHSe reduction. Results suggest that interventions for reduction in ETS may be more successful than for smoking cessation and relapse prevention in families of children aged less than 5 years. There is a need for a range of interventions to support families in creating a smoke free home environment that are both tailored and targeted to specific populations. Interventions that target the social and psychodynamics of the family should be considered further, particularly in reaching vulnerable populations. Consideration is also required for approaches to interventions that may further stigmatise families containing smokers. Further research is required to identify successful elements of interventions and the contexts in which they are most effective.

## 1. Introduction

Tobacco smoking in Western countries has declined in response to a range of policy, health promotion and education initiatives. While the prevalence of smoking in Western developed countries is now generally less than 20% in adults [1], people who continue to smoke include those in families with infants and children.

Exposure to adult smoking presents several risks to children. The World Health Organisation (WHO) estimates that one third of premature deaths attributable to environmental tobacco smoke (ETS) occur in children and that ETS contributes to the premature death of approximately 1100 children with asthma per annum [2]. Environmental tobacco smoke includes not only secondhand smoke exposure (SHSe) through passive exposure to tobacco smoke, but also thirdhand smoke exposure (THSe), via exposure to the toxic contaminants of tobacco smoke that remain in the environment particularly on clothing, hair and surfaces [3,4]. Where smoke-free legislation has been introduced, there has been a clear and corresponding decrease in preterm births and hospital admissions for asthma [5]. In addition to the physical risks from adult tobacco smoking, there are risks to children in the forms of behavioural effects of smoking in that children who have parents or siblings who smoke are more likely to smoke themselves [6,7,8,9] and to begin at an earlier age [10]. If both parents and siblings smoke, the risk of smoking is greater still [6,11].

Although smoking most commonly begins during adolescence, even young children recognise and respond to observed smoking behaviours. By the time children start school, they have begun to understand tobacco use. For example, at 5 years of age, children can recognise and identify cigarettes [12] and, in role play, demonstrate an awareness of how adults obtain and use tobacco [13,14]. By age 9, children can begin to identify reasons why someone may choose to smoke, including image, role modelling, stress relief and mood enhancement [15]. This suggests that parental role modelling of smoking is influential in children’s views and beliefs, even when children are aware of detrimental health effects and that interventions with parents and families in the early years of childhood may be important to children’s views and beliefs about smoking [15].

Concerns about the impact of smoking on young children have led to the development of interventions to assist families with harm minimisation including smoking cessation, ETS reduction, and antismoking socialisation. Antismoking socialisation has been defined as parenting behaviours and interactions that influence children’s cognitive and behavioural responses against smoking [16]. Parents’ behaviours and interactions may include communication about the risks of smoking, the setting of rules around smoking both for themselves and their children, monitoring of children’s behaviour and other methods of socialisation. Such interventions are important, as family is the first smoking socialisation context for children and young people. It is within the context of family that parents can positively or negatively influence children’s health behaviours [17].

There is evidence that smoking is associated with socioeconomic disadvantage and lower education and income [18,19]. As an example, single parent mothers are twice as likely to smoke as mothers living with a partner [20]. Almost half (47%) of Australian Indigenous people aged 15 years and older report being current smokers, compared with 17% of the broader Australian population [21]. Current smokers are more likely than non-smokers to be dealing with emotional and social difficulties, including psychological distress [22,23] and racial discrimination [23].

As such, a socio-ecological framework may provide a useful tool for organising and addressing these influencing agents from different environmental spheres [24]. Implicit in the model is an assumption that individual health behaviour is influenced by both individual beliefs and values as well as the beliefs and values of the individuals’ primary social groups, their social and community institutions and networks, and public policy [24]. These multiple levels of influence include intrapersonal (e.g., age, gender, knowledge, behaviour, self-efficacy, skills), interpersonal (personal networks, such as family, workplace and friends), institutional factors (e.g., neighbourhood, practices and policies of workplace, child care), community (community norms, relationships between organisations and institutions), and public policy (local and national laws and regulations).

Factors across the levels of the socio-ecological framework need consideration when developing interventions for smoking abstinence, cessation, and socialisation. However, they have been largely ignored by previous literature reviews [25,26,27]. One review assessed interventions designed to support families in their efforts to promote non-smoking in children [28], but excluded studies where the parent intervention was not tested separately to the other parts of the intervention. A more holistic approach is needed to understand what levels and components of interventions are most effective.

### Objectives

The purpose of this review was to describe the state of the science of family-focussed interventions to promote smoke-free home environments for infants and children under 5 years, including parent smoking reduction and cessation interventions, SHSe reduction, and anti-smoking socialisation interventions, using the socio-ecological framework as a guide. All interventions that planned to intervene with families to support parent smoking cessation or reduction, or reduce ETS in the home or any other targeted program aimed at families of children aged 0–5 years were included. The outcome measures included any changes in the smoking behaviour of families, including smoking cessation or reduction, household restrictions on smoking, knowledge, attitudes and beliefs about smoking, child smoking behaviour (longitudinal), exposure to ETS (including biochemical measures and parent reported exposure), child health outcomes (illness events, respiratory symptoms, change in lung function, utilization of health care services). Studies published from 2000 to 2014 were included to ensure that the most contemporary research relevant to the current context of interventions in smoking cessation and harm reduction was captured.

## 2. Methods

### 2.1. Protocol

This review was guided by current methods for systematic searching and selecting evidence for a literature review [29,30].

### 2.2. Eligibility Criteria

Papers were included if they were: (1) empirical study reports of interventions aimed at smoking cessation, promoting a smoke free home environment or antismoking socialisation; and (2) focused on primary carers (parents, guardians, foster carers or grandparents) involved in the parenting of infants and young children and/or young children. Where child age range exceeded 0–5 years, a mean age within the 0–5 year range was used as a criterion. Included papers were published between 2000 and 2014 in peer reviewed journals to ensure a focus on the most recent research in the topic. Papers were excluded if they were not written in English.

### 2.3. Information Sources

Electronic databases searched included MEDLINE, Cochrane Database of Systematic Reviews, PubMed, and CINAHL. Search terms included cigarettes, smoking, tobacco, parent, and family, as well as terms aimed at identifying intervention studies (An example appears in Table 1). The reference lists of included studies were searched manually.

**Table 1:** Medline search strategy.

**Term set 1:** Child *
**Term set 2:** Parent * OR father * OR mother * OR caregivers OR famil * OR school * OR communit *
**Term set 3:** Cigar * OR tobacco * OR smok * OR smoking cessation OR tobacco cessation OR tobacco smoke pollution OR smoking abstinence
**Term set 4:** prevent * OR control *
**Term set 5:** intervention OR clinical trial OR pilot study OR outcomes OR randomised control trial
**Term set 6:** 1 and 2 and 3 and 4 and 5

### 2.4. Study Selection

All literature identified from the electronic searches were imported into the Endnote Reference Management System version 5. The title and abstract of each study were reviewed against the inclusion criteria, with full text being reviewed as required.

### 2.5. Data Collection Process and Data Items

Data were extracted using a standardised form. Data included country, intervention setting (e.g., community health, acute health care service, school, preschool), participants (demographic information), intervention details, and primary and secondary outcomes for the study. In accordance with Preferred Reporting Items for Systematic reviews and Meta Analyses (PRISMA) guidelines [30] and critiques of the reporting of interventions for behaviour change [31], details were extracted for each intervention by one of the reviewers (NB), including content, delivery personnel, method of communication, intensity, complexity, environment and conceptual framework. Any concerns about the nature of the articles selected were discussed in conjunction with a second reviewer (TL).

### 2.6. Risk of Bias

The quality of the included studies was assessed by the first author using the United States Preventative Services Taskforce (USPST) procedures for critical appraisal of research [32]. USPST procedures include appraisal of the research design, internal and external validity, study population, location and provider (Table 2).

### 2.7. Synthesis of Results

The main aim of the literature review was to appraise and synthesize evidence across a broad range of interventions with families using the framework of the socio-ecological model. It was anticipated that there would be considerable heterogeneity of study aims, designs, methods and outcomes and that existing systematic reviews would be included, and thus narrative synthesis rather than meta-analysis was used to guide data synthesis. The synthesis followed a combination of methods recommended by Popay and colleagues [29], including tabulation and content analysis. These guidelines were developed to facilitate narrative synthesis in systematic reviews where the effectiveness of interventions and the factors influencing the implementation of interventions are central [33].

## 3. Results

**Figure 1:**
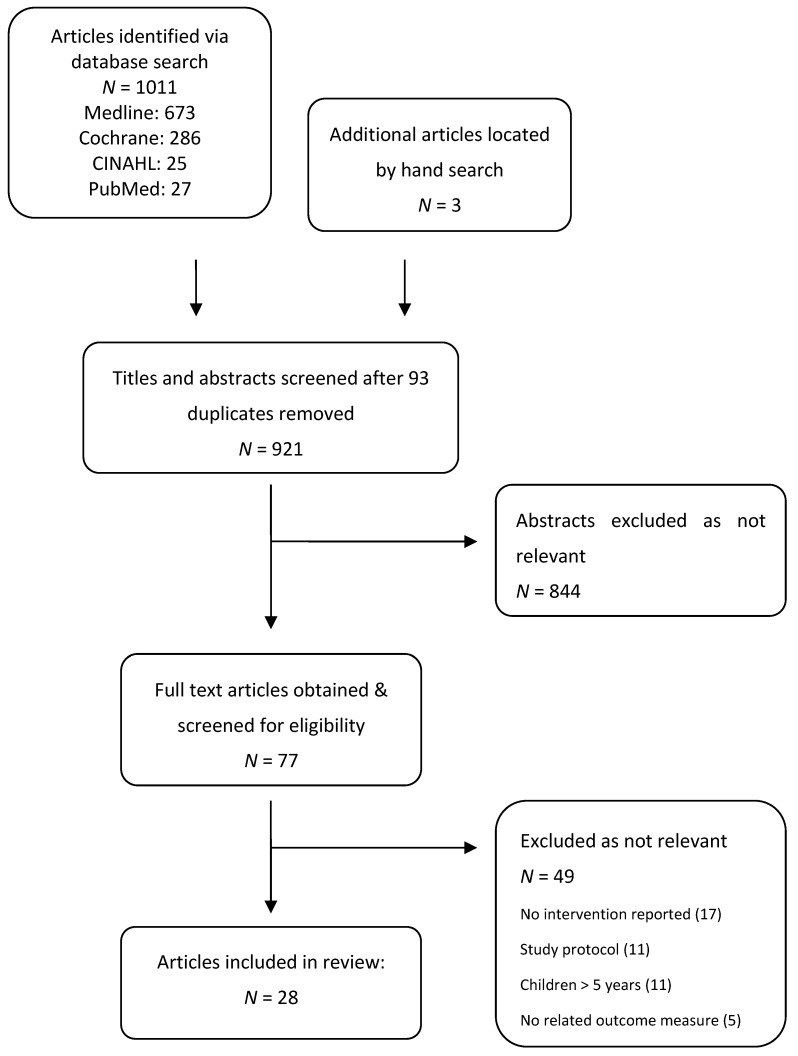
Search strategy.

The initial search located 921 articles following removal of duplicates (Figure 1). After review against inclusion criteria, 28 articles were included including smoking cessation (*n =* 15), ETS reduction (*n =* 12) and anti-smoking socialisation interventions (*n =* 1).

The studies were assessed for quality against USPSTF methods, and were categorised as good, fair or poor (Table 3). The majority of studies were fair quality, with only two of the studies rated as good [34,35]. The main concerns with studies rated as fair or poor were related to limitations with randomisation or allocation concealment encountered in intervention design and delivery.

### 3.1. Smoking Cessation Interventions

Fifteen articles on smoking cessation were reviewed and, of these, two articles were drawn from the same study [36,37]. The majority were from the United States and Europe and used a prospective single centre randomised controlled trial design (Table 3).

#### 3.1.1. Target Populations

Most studies targeted families in the postpartum period. Of these, five studies were designed to prevent relapse in parents who had stopped smoking in response to pregnancy, or to encourage smoking behaviour change or cessation in parents who were still smoking [35,38,39,40,41]. One study specifically targeted parents of infants at high risk for severe asthma [36,37]. Only two studies reported on family based interventions of children aged 1–5 years [42,43]. Studies varied considerably in sample size–from 31 to 3889 (Table 3).

**Table 2:** Study design and level of quality (AHRQ 2008).

Reference	Focus	Design	Internal Validity	External Validity
Phillips *et al.* 2012. USA [35]	Smoking relapse prevention	RCT	Good	Good
Hovell *et al.* 2009. USA [34]	Smoking cessation/SHS reduction	RCT	Good	Good
Kuiper *et al.* 2005. Schonberger *et al.* 2005. Netherlands [36,37]	Smoking cessation/SHS reduction	RCT	Fair	Good
Chan-Yeung *et al.* 2000; Becker *et al.* 2004, Chan-Yeung *et al.* 2005. Canada [55,56,57]	SHSe reduction	RCT	Fair	Good
Conway *et al.* 2004. USA [59]	SHSe reduction	RCT	Fair	Good
Joseph *et al.* 2014. USA [43]	Smoking cessation	Pilot Quasi-experimental	Fair	Fair
Jiminez-Muro *et al.* 2013. Spain [38]	Smoking cessation/relapse prevention	RCT	Fair	Fair
Storrø *et al.* 2010. Norway [42]	Smoking reduction	Cohort control trial with one year time difference	Fair	Fair
Winickoff *et al.* 2010. USA [40]	Smoking cessation/reduction	Quasi RCT	Fair	Fair
Hannover *et al.* 2009. Germany [39]	Smoking cessation/relapse prevention	Quasi RCT	Fair	Fair
Kallio *et al.* 2006. Finland [46]	Smoking cessation/reduction/SHS reduction	RCT (longitudinal)	Fair	Fair
Abdullah *et al.* 2005. Hong Kong [45]	Smoking cessation	RCT	Fair	Fair
Wiggins *et al.* 2005. UK [47]	Smoking cessation	RCT	Fair	Fair
Baheiraei *et al.* 2011. Iran [53]	SHSe reduction	RCT	Fair	Fair
Emmons *et al.* 2001. USA [52]	SHSe reduction	RCT	Fair	Fair
Kitzman *et al.* 2010. USA [61]	Smoking prevention	RCT (longitudinal)	Fair	Fair
Øien *et al.* 2008. Norway [44]	Smoking cessation	Control trial	Fair	Poor
Culp *et al.* 2007. USA [48]	Smoking cessation	Quasi-experimental	Fair	Poor
Wilson *et al.* 2013. Scotland [54]	SHSe reduction	Pilot RCT	Fair	Poor
Huang *et al.* 2013. Taiwan [60]	SHSe reduction	RCT	Poor	Fair
Harutyunyan *et al.* 2013. Armenia [50]	SHSe reduction	RCT	Poor	Fair
Fossum *et al.* 2004. Sweden [58]	SHSe reductions	CT	Poor	Fair
Zakarian *et al.* 2004. USA [51]	SHSe reduction	Quasi-experimental	Fair	Poor
Disantis *et al.* 2010. USA [41]	Smoking cessation/relapse prevention	Pilot 2 arm experimental	Poor	Poor
Yücel *et al.* 2014. Turkey [49]	SHSe reduction	RCT	Poor	Poor

**Table 3:** Study designs and outcomes.

Reference	Focus	Participants	Design	Outcomes/Results
Joseph *et al.* 2014. USA [43]	To investigate feasibility of screening serum cotinine with lead screening to increase parental smoking cessation and implementation of home smoking restrictions.	80 smoking parents of children at well child clinics for 12 and 24 month checks.	Pilot Quasi-experimental	**Parent smoking cessation:** 74% engaged in smoking counselling and 24% accessed NRT. 7 day point prevalence abstinence at 8 weeks: IG 11/40 (29%) *vs.* CG 1/40 (*p* = 0.001).
				**Home smoking restrictions:** High levels of smoking restriction at baseline in both groups, change not significant (IG full ban: 67.5% at baseline *vs.* 86.8% at 8 weeks; CG full ban 77.5% at baseline *vs.* 80% at 8 weeks).
Jiminez-Muro *et al.* 2013. Spain [38]	To analyse the efficacy of a motivational interview intervention in postpartum women to prevent relapse in recent quitters and encourage behaviour change in those still smoking.	412/626 postpartum women smokers. 64% Spanish, 34% immigrants.	RCT	**Continuous abstinence:** Probability of remaining abstinent at 12 week was 74% (IG) & 37% (CG) (*p* < 0.001).
				**Urine Cotinine:** Only 49% of participants attended 3 month visit and therefore biochemical validation was not statistically significant (int 31%, control 23%, n.s.).
Phillips *et al.* 2012. USA [35]	To reduce smoking relapse and prolong breastfeeding in mothers during the first 8 weeks postpartum.	54 mothers of an infant in NICU. Mothers had a history of tobacco use during or within one year of pregnancy, but currently not smoking.	RCT	**Maternal smoking status at 8 weeks postpartum:** Significant decrease in smoking relapse at 8 weeks postpartum in the int gp (IG: 81% *vs.* CG: 46%, *p* < 0.001).
				**Salivary cotinine:** A 94% agreement was found between salivary cotinine level and mothers reported smoking status.
Disantis *et al.* 2010. USA [41]	To pilot a postpartum smoking intervention that combined postpartum smoking cessation & relapse prevention with breastfeeding counselling.	31 low income women who were either current smokers or recent ex-smokers. Hispanic (50%), African-American (25%). Primiparous (45.8%). 62.5% completed high school or higher education. Years of smoking M = 6.96 years (SD = 5.67). Daily cigarettes M = 12.5 (SD = 7.7) 51% quit smoking prior to pregnancy.	Pilot 2 arm experimental	**7-day point prevalence:** S + B: 50%; RP: 75%, not significant.
				**Days to relapse:** related to duration of breastfeeding (*r* = 0.92, *p* = 0.08).
				S + B: mothers who quit before or during pregnancy had higher rates of smoking abstinence than those who smoked through pregnancy (x^2^ = 4.00, *p* < 0.05).
Storrø *et al.* 2010. Norway [42]	To evaluate the impact of a primary prevention intervention program on risk behaviour for allergic disease in primary health care settings (increase cod liver and oily fish intake, reduce parental smoking, reduce indoor dampness).	2860 pregnant women or women with a child <2 years of age.	Cohort control trial with one year time difference	**Maternal smoking frequencies:** Significant and stable decline in smoking from pregnancy to 2 years postnatal, not attributable to intervention. In addition, there was a statistically significant annual trend in the control cohort. (**Baseline:** IG 17.3% *vs.* CG 23.6%. OR 0.70, 95% CI 0.60-0.82. **6 weeks:** IG 5.3% *vs.* CG 10.8%. OR 0.55, 95% CI 0.42-0.70. **2 years:** IG 9.9% *vs.* CG 19%. OR 0.50, 95% CI 0.41–0.61).
Winickoff *et al.* 2010. USA [40]	To test an intervention to address maternal and paternal smoking during postpartum hospitalization.	101/173 parents. 71% current smokers, 29% recent quitters. 67% female.	Quasi RCT	**7 day point prevalence of cotinine verified tobacco abstinence for 3 months:** Self-reported 7 day abstinence not significant (IG: Decreased 31% to 25%; CG: 38% to 28%. Effect 9.4%, n.s.). Cotinine confirmed 7 day abstinence rate at follow up IG: 9% *vs.* CG: 3% (n.s.).
				**Self-reported 24 h quit attempts:** IG: 64%; CG: 18%, *p* = 0.005.
Hannover *et al.* 2009. Germany [39]	To test the efficacy of an intervention to aid cessation/relapse prevention for postpartum women.	644 women from 6 hospitals with postpartum units.	Quasi RCT	**Sustained abstinence (Still not smoking at 6 months or since birth):** No statistically significant difference at follow up.
				**Repeated 4 week point prevalence (not smoking 4 weeks prior to follow up)**.
				**No statistically significant difference in sustained abstinence at either follow up. Statistically significant 4 week point prevalence abstinence at 6 months only**.
Hovell *et al.* 2009. USA [34]	To test the effects of SHS and smoking counselling in high risk families.	150/244 mothers of children aged less than 4 years exposed to minimum of 3 maternal cigarettes per day.	RCT	**Reported SHS exposure:** Decrease in both IG (80%) & CG (55%) in first 6 months. Group main effect 6–18 months significant for IG (*p* = 0.011).
				**Child urine cotinine:** Decreased baseline to 6 months only (25% both gps). Only the group main effect significant for 6–18 months (*p* = 0.026). Controls higher throughout baseline & follow up.
				**Maternal smoking (self-report):** 6 months: IG decreased by 34%, CG decreased 5%. 6–18 months: IG decreased by 33% CG, 4.6%.
				**Smoking cessations:** 17% IG and 5.4% CG quit smoking for 7 days before one or more study measures.
Øien *et al.* 2008. Norway [44]	Investigate parental smoking behaviour during pregnancy after introduction of a prenatal, structure, multidisciplinary smoking cessation intervention.	3839 pregnant women attending primary health care settings. Estimated participation rate of 44% of eligible women in the location (Tondheim). Low smoking prevalence at inclusion (IG: 4.9%, CG: 7.1%).	Control trial	**Self-reported smoking behaviour 6 weeks postnatal**. No significant difference between IG and CG.
Culp *et al.* 2007. USA [48]	Evaluate health and safety intervention with first time mothers.	355 pregnant women in rural south-western states (IG: *n =* 156, CG: *n =* 107). 61% smokers.	Quasi-experimental	**Maternal smoking behaviour (no. of cigarettes/day):** Baseline: n/s between IG and CG. Six months: IG smoking 2.4 fewer cigarettes per day (IG: M = 6.34; SD = 6.95 *vs.* CG: M = 8.72, SD 7.26, *t* (147) = 2.0, *p* = 0.023). Twelve months: IG smoking 2.1 fewer cigarettes per day (IG: M = 7.28, SD = 6.79 *vs.* CG: M = 9.41, SD = 7.09) *t* (147) = 1.82, *p* = 0.071.)
				**Knowledge of the effects of smoking on child development:** e.g., Impaired brain development (IG 59.2% *vs.* CG 41.7%, *p* ≤ 0.01); lower mental health scores (IG 52.6% *vs.* CG 32.3%, *p* < 0.001).
Kallio *et al.* 2006. Finland [46]	To determine whether repeated lifestyle counselling alters parental smoking and child exposure to tobacco smoke.	1062/1105 parents of infants attending a well baby clinic.	RCT (longitudinal)	**Parent smoking:** Decreased across IG and CG over time. No significant difference between groups.
				**Serum cotinine of children:** 46% of 8 year olds had been exposed to nicotine in last few days. None had high enough levels to confirm that they had smoked. Serum cotinine highest in children with both parent smokers. Serum cotinine higher in families where only father smoked than where only mother smoked. 24% of children from non-smoking families had cotinine higher than 1 ng/mL.
Abdullah *et al.* 2005. Hong Kong [45]	To evaluate whether telephone counselling based on stages of change could help non-motivated smoking parents of young children to cease.	952 smoking parents of Chinese children aged 5 years (85.3% fathers).	RCT	**7 point prevalence quite rate at 6 months:** Higher in IG (15.3%: 68/444) than CG (7.4%: 34/459) *p* < 0.001. Absolute risk reduction 7.9% (95% CI: 3.78% to 12.01%). Number needed to treat 13 (95% CI: 8–26).
Kuiper *et al.* 2005. Schonberger *et al.* 2005. Netherlands [36,37]	To evaluate a multifaceted intervention strategy to reduce occurrence of severe asthma (smoking cessation, SHSE avoidance, dust mite avoidance, breastfeeding, timing of introduction of solid food).	Parents of 476 infants at high risk of severe asthma.	RCT	**Self-report of SHSe at one year:** No data reported. Authors state “No difference was found in the intervention compared with the control group concerning the exposure to tobacco smoke” (p. 329).
				**CO monitoring:** No results reported.
Wiggins *et al.* 2005. UK [47]	To evaluate the effect of two forms of postpartum social support (support health visitor (SVH) or community group support (CGS) on maternal and child health outcomes (maternal smoking).	731 women with infants from culturally diverse and disadvantaged inner city areas of London. Approx 26%–30% smokers across groups. 14% non-English speakers.	RCT	**Maternal smoking:** not significantly reduced (SVH *vs.* CG: 95% CI 0.86 (0.62, 1.19); CGS *vs.* CG: 95% CI 0.97 (0.72, 1.33).
Yücel *et al.* 2014. Turkey [49]	To evaluate the effectiveness of an intensive intervention *vs.* a minimal intervention to reduce SHSe.	Parents of 182 children aged 1–5 years.	RCT	**Urinary cotinine–pre and post intervention:** Urine cotinine decreased across time in both groups. Decrease greater in intensive IG than minimal IG, but n.s. (*p* = 0.831).
				**Complete home smoking bans:** Authors report that 30.6% of Intensive IG households who did not have a ban at baseline, did have a total ban at 3 months (*p* = 0.001). In the minimal IG, 10.5% more families had ban at 3 months, but n.s (*p* = 0.125).
Wilson *et al.* 2013. Scotland [54]	To investigate feasibility of an intervention (REFRESH) to reduce SHSe for children in their homes.	59/1693 smoking mothers with at least one child younger than 6 years. Maternal age M = 30 years; child age M = 3.5 years (range 1.2–5.7 years).	Pilot RCT	**Difference in PM_2.5_ from visit 2 to visit 4:** Greater reduction achieved for maximum PM.
				**Peak concentration of PM_2.5_:** IG 67 *vs.* CG 148 (*p* = 0.006).
				**The percentage of time when household PM_2.5_ concentrations exceeded 35 μ/m^3^:** IG 0.49 *vs.* CG 3.6 (*p* = 0.017).
				**Children’s salivary cotinine:** No significant difference.
				**Feasibility, acceptability and understanding of intervention:** Qualitative data–intervention was acceptable and mothers were able to understand the data.
				**Motivators and mechanisms of change:** Personalised data made the concept of the dangers of SHSe more real to them and mothers reported a greater sense of motivation for change.
Huang *et al.* 2013. Taiwan [60]	To evaluate the effectiveness of a transtheoretical model- based passive smoking prevention program for pregnant women and mothers of young children.	294/335 women recruited from obstetrics and paediatric departments of four hospitals. IG: 48% pregnant. CG: 45% pregnant. Remainder mothers of children aged <3 years.	RCT	**Stages of change:** 73% were already in target stage at baseline. Less than 30% of the remaining changed stage. Distribution of stages of change statistically different after intervention between participant groups (mothers with children: F = 11.978, *p* = 0.003; pregnant women: *F* = 6.689, *p* = 0.035).
				**Knowledge:** No significant difference between groups pre or post test.
				**Frequency of avoiding passive smoking:** Significant difference in intervention group (*F =* 5.115, *p* = 0.25) at post-test.
				**Self-efficacy:** No significant difference.
Harutyunyan *et al.* 2013. Armenia [50]	To test an intense intervention to reduce child SHSe.	250 households with children aged 2–6 years recruited via paediatrician primary health care clinics.Maternal age M = 30 years (SD 5.2 years). 53% employed, 36% had a university degree. Household smokers predominately fathers (80%). Child age M = 4 years (SD 1.2 years). Smoking was permitted in all households, some restrictions in approximately half of homes.	RCT	**Child hair nicotine concentration:** 17% lower in IG than CG although not significant (*p* = 0.239). Significantly decreased in IG from baseline to follow up (0.30 ng/mg to 0.23 ng/mg; *p* = 0.77).
				**Maternal knowledge of SHSe and smoking hazards:** IG: From 9.5 at baseline to 11.3 at follow up. CG: From 9.8 to 10.5. 10% higher in IG than CG after controlling for baseline score (*p* = 0.006).
Baheiraei *et al.* 2011. Iran [53]	To assess whether counselling both mother and father reduces infant SHSe.	130 parents of health infants (<12 months) with at least one parent smoker. Families from predominately lower SES.	RCT	**Urine cotinine:** Decreased for both groups but significantly decreased in IG (Baseline: IG 48.72 *vs.* CG 40.83; 3 months IG: 28.68 *vs.* CG 3.32). *p* = 0.029).
				**Total daily cigarette consumption:** Greater decrease in presence of child in IG (media*n =* 0, interquartile range: 0, 2.71) than CG (media*n =* 1, interquartile range: 0, 3.21) at the 3 month follow up (one tailed *p*, 0.3). No significant correlation between cigarettes consumed and reported level of SHSe.
				**Home and car smoking bans:** Increase in both IG & CG, but not significant in CG. Statistically significant between groups (*p* = 0.49).
Fossum *et al.* 2004. Sweden [58]	To evaluate the effects of a counselling intervention (Smoke Free children).	41 mothers of newborn infants attending child health clinics.	CT	**Self reported smoking:** More IG mothers reported smoking at baseline (M = 13.1, SD 6.5 than CG (M = 10.8, SD 5.7) and after intervention (M = 12.8, SD 5.9) than CG (M = 8.2, SD 4.3).
				**Maternal saliva cotinine:** Cotinine levels increased by 40% in CG and decreased by 10% in IG (*F* = 5.501, *df* = 1, *p* = 0.027).
Zakarian *et al.* 2004. USA [51]	To evaluate the effectiveness of a behavioural counselling program for reducing child SHSe.	150 mothers of children aged less than 4 years attending a well-child community clinic. Most mothers were White, not employed, low education. Approximately 40% were single parents.	Quasi-experimental	**Maternal report of child SHSe (number of maternal cigarettes child exposed to per week:** Declined for baseline to 6 months post-test for both groups (IG: 18.89 at baseline to 5.41 at 12 months. CG: 13.25 at baseline to 5.23 at 12 months) (*p* < 0.001). Data presented in graph difficult to report exact results. Priest *et al.* (2008) reported data. Total exposure to cigarettes/week (IG 53.2 at baseline to 21.99 at 12 months. CG: l 54.48 atbaseline to 18.22 at 12 months) (*p* < 0.001).
				No significant group x time differences. Number of counselling sessions completed was not a significant covariate.
				**Children’s urinary cotinine concentration:** No significant change over time in either group. No significant group x time or group differences.
				**Maternal smoking rates:** Similar to SHSe above, a sharp decline from baseline to post-test across both groups.
				**Maternal smoking cessation:** Self-reported 7-day quit status did not vary by experimental group at any time point.
Chan-Yeung *et al.* 2000; Becker *et al.* 2004, Chan-Yeung *et al.* 2005. Canada [55,56,57]	Prevention of asthma in high-risk infants via multifaceted intervention program (house dust mite control, pet avoidance, avoidance of ETS, promotion of breastfeeding).	545 infants at high risk for asthma and their families. 7% of mothers smoking at baseline (36/493).	RCT	**Parental smoking cessation:** No significant difference in proportion of mothers, fathers or others who gave up or acquired smoking at 12 months.
Conway *et al.* 2004. USA [59]	To evaluate the effectiveness of a lay delivered intervention to reduce ETS exposure in Latino children.	143 Latino parent-child pairs. Child age 1–9 years (M = 4 years).	RCT	**Child hair nicotine (log ng/mg):** Baseline (IG: 0.25 *vs.* CG 0.23), post intervention (IG: 0.17 *vs.* CG: 0.19, 3 months (IG: 0.28 *vs.* CG 0.32), 12 months: (IG: 0.23 *vs.* CG: 0.23). No significant differences between groups over time.
				**Child hair cotinine (log ng/mg):** Baseline (IG 0.05 *vs.* CG 0.05), post intervention (IG 0.03 *vs.* CG 0.03), 3 months (IG 0.04 *vs.* CG 0.04), 12 month (IG 0.02 *vs.* CF 0.04). No significant differences between groups, but time effect detected (*p* < 0.001).
				**Parent report of number of cigarettes child exposed to in household over one month:** Baseline (IG 1.75 *vs.* CG 1.85), post intervention (IG 1.42 *vs.* CG 1.62), 3 months (IG: 1.27 *vs.* CG 1.44), 12 months (IG: 1.06 *vs.* CG 1.27). No significant difference between groups, trending toward significance over time (*p* = 0.048).
				**Confirmed reduction (dichotomous variable based on parent report and child hair biomarkers:** Not significant.
Emmons *et al.* 2001. USA [52]	Outcome evaluation of project KISS (Keep Infants Safe From Smoke).	291 smoking low-income parent/caregivers. Children younger than 3 years.	RCT	**Nicotine levels in household:** significant time-by-treatment effect (*F* (2406) = 4.80, *p* < 0.01). IG: Levels at 3 & 6 months significantly lower than baseline (*F* (2200) = 4.36; *p* < 0.5).
				**Smoking cessation:** Overall cessation 7.5% CG *vs.* 10.1% IG. No significant difference between groups.
Kitzman *et al.* 2010. USA [61]	To test the effect of prenatal and infancy home visits by nurses on 12 year old first born children’s use of substances (cigarettes, alcohol, marijuana).	1139 low SES African-American women pregnant with first child.	RCT (longitudinal)	**Substance use by children:** IG less likely to have used substances (CG: 5.1 *vs.* IG 1.7, OR 0.31, *p* = 0.04), to have used fewer of these substances (incidence ratio = 0.22, *p* = 0.02) and to have used these substances for fewer days (incidence ratio, 0.15, *p* = 0.02).

CG: Control group, IG: Intervention group, NRT: Nicotine replacement therapy, PM_2.5_: Airborne particulate matter < 2.5 μm in size, RR: Response rate, SC: Standard care; SES: Socioeconomic status, SHSe: Second-hand smoke exposure, ETS: Environmental tobacco smoke, UK: United Kingdom, USA: United States of America.

#### 3.1.2. Interventions

The content and focus of interventions ranged considerably (Table 4). Four studies reflected existing smoking cessation intervention practice guidelines or programs [40,42,44] or smoking cessation information tailored to stages of change [45]. Two studies used education relating to healthy behaviours and risk of smoking [38,46]. Two studies had no direct intervention that focussed on smoking or associated risk at all. Instead, the focus was on the promotion of bonding and attachment between the parents and newborn infant as a way to promote smoking cessation [35] or through different models of social support during the early postpartum period [47]. A further three studies included smoking cessation interventions within the context of a universal health promotion program [46,48] or as one part of a multifaceted intervention to reduce the risk of severe asthma in at risk infants [36,37].

In most instances, the intervention was delivered either by research personnel who had received additional training in smoking cessation [36,37,38,39,40] or health care professionals [42,44,45,47]. Most interventions took place in an individual face to face counselling session. Some studies augmented these sessions with phone counselling [39] or with written or audio-visual materials [35,38].

There was considerable variation in the intensity and duration of interventions. They ranged from brief, single interventions [40] to a repeated intervention over a seven year period [46]. Interventions took place either in the home or a clinical environment.

Limited detail of the conceptual frameworks underpinning interventions was provided in the retrieved studies. Those that did provide details had utilised the principles of motivational interviewing [38,39,43], the 5A model for smoking cessation [40,42] or the transtheoretical model of behavioural change [45]. In the two studies where the intervention did not focus on smoking as a risk, the intervention designs suggested that attachment theory [35] or social support [47] were used.

#### 3.1.3. Outcome Measures

All studies used primary outcome measures that were based on self-report of smoking abstinence status such as 7-day point prevalence [40,41,43], self-report of smoking status at a time point [35,44,46,47,48], or self-report of continuous smoking abstinence [38,39] (Table 3). Four studies used biochemical measures as a secondary outcome to verify the self-report measures including maternal urine cotinine [38,40], maternal salivary cotinine [35], or cotinine measures from the parent’s children [46]. Carbon monoxide monitoring [36,37] was used, but results were unreported. Additional secondary outcomes included home smoking restrictions or bans [43] and maternal knowledge of second hand smoke effects [48].

#### 3.1.4. Effectiveness

Of the 13 studies reviewed, only four reported statistically significant positive effects [35,38,43,45].

**Table 4:** Characteristics of interventions.

Author	Content	Delivery Personnel	Method of Communication	Intensity/Complexity	Environment	Conceptual Framework	Socio-Ecological Model
Smoking cessation/relapse prevention																		
Joseph *et al.* 2014 [43]	Serum cotinine feedback, SHSe education, optional counselling, optional NRT	Trained tobacco advisor	Mail and phone	Weekly for 8 weeks	Home	MI, CBT	Intrapersonal
Jiminez-Muro *et al.* 2013 [38]	Risks of smoking, health behaviours	Research student	Phone	5 × 15 minute calls over 3 months	Home (phone)	MI	Intrapersonal
Phillips *et al.* 2012 [35]	Newborn cues	Not stated. Partially self-administered	DVD Brochure	Not described	Hospital and home	Attachment theory	Intrapersonal
Disantis *et al.* 2010 [41]	Smoking and breastfeeding counselling OR relapse prevention	Counsellor	Face to face Written materials	15 minutes + written materials	Clinic	Not stated	Intrapersonal
Storro *et al.* 2010 [42]	Brief 5As	GP or midwife	Face to face	At least 5 occasions	Clinic	Brief 5As	Intrapersonal Interpersonal
Winickoff *et al.* 2010 [40]	Brief 5 As	Trained study staff	Face to face	15 minutes + offer to enroll in Quitline	Hospital	Brief 5As	Intrapersonal Interpersonal
Hannover *et al.* 2009 [39]	Relapse prevention/smoking cessation counselling	Trained study staff	Face to face + phone	Single interview + phone follow up × 2	Home	MI	Intrapersonal
Hovell *et al.* 2009 [34]	SHSe reduction and tailored smoking cessation including option of NRT	Study counsellor	Face to face + phone	14 sessions over 7 weeks. Mean time/session: 23 minutes	Home	Learning theory	Intrapersonal Interpersonal
Oien *et al.* 2008 [44]	Brief office intervention (Fiore *et al.* 2000)	Midwives, GP, nurses	Face to face	Not clear	Primary health care	Not stated	Intrapersonal
Culp *et al.* 2007 [48]	Universal program, including smoking and effect of SHSe on infant growth and development	Visitors with child development degree level qualifications	Face to face	Average 10.9 visits before birth + 20.7 visits after birth (approx 1 h per visit)	Home	Not reported	Intrapersonal
Kallio *et al.* 2006 [46]	Universal program including smoking	Paediatrician and dietician	Face to face	Paediatrician: every 1–3 months until 2 years Dietician: every 4–6 months until 2 years. Dietician and paediatrician every 6 months until 7 years	Clinic	Not reported	Intrapersonal
Abdullah *et al.* 2005 [45]	Smoking cessation and SHSe reduction tailored to stage of change. No NRT information	Nurse	Phone + written materials	Three phone calls × 20–30 min	Home via phone	Transtheoretical model (stages of change)	Intrapersonal
Kuiper *et al.* 2005. Schonberger *et al.* 2005 [36-37]	Smoking cessation and home bans on smoking	Research nurse	Face to face	Once	Not explained	Not explained	Intrapersonal Interpersonal
Wiggens *et al.* 2005 [47]	Social support	Health visitor OR non-professional	Face to face	1.5–10 h	Home OR community centre	Not explained. ? social support	Intrapersonal Interpersonal
**SHSe reduction interventions**																			
Yucel *et al.* 2014 [49]	SHSe information, goal setting, use of resources, urine cotinine feedback	Researcher	Face to face Phone Written materials	Intensive group: Home visits at baseline, 1 & 3 months. Phone calls at 6 & 8 weeks. Minimal intensity group: Home visit at baseline and 3 months. Mail out urine cotinine result	Home	Not stated	Intrapersonal
Wilson *et al.* 2013 [54]	24 h measure on home air quality PM_2.5_ (particulate matter) & motivational interview	Research staff	Face to face	Four visits over a one month period	Home	MI	Intrapersonal
Huang *et al.* 2013 [60]	Impact of passive smoking, avoiding passive smoke in public and at home. Sections tailored to stages of change.	Research staff	Face to face, audiovisual, written materials, phone	Time not stated. Included DVD, booklet, stickers, phone follow up at 2 weeks and 3 weeks post intervention	Home	Transtheoretical model (stages of change)	Intrapersonal
Harutyunyan *et al* [50]*.*	Importance of healthy environment, dangers of smoking and SHSe, smoking cessation, smoke-free home, PM_25_ feedback, written materials. CG: written materials only	Research staff	Face to face Written materials Phone	40 minute MI + 2 follow up phone calls (timeframe not specified)	Home	MI	Intrapersonal Interpersonal
Baheiraei *et al.* 2011 [53]	Smoke free children (Fossum *et al.* 2004 [58])	Research student	Face to face Phone Written materials	One face to face interview + two phone interviews (max. 20 min each)	Home	MI	Intrapersonal
Chan-Yeung *et al.* 2000, Becker *et al.* 2004, Chan-Yeung *et al.* 2005 [55,56,57]	Counselled on smoking cessation and instructed to keep house smoke free	Research nurse	Face to face	Single prenatal visit	Home	Risk factors for asthma	Intrapersonal Interpersonal
Conway *et al.* 2004 [59]	Problem solving aimed at lowering child ETS in the household	Lay bicultural and bilingual Latina community health advisors. All received 20 h training over 4 weeks	Face to face Phone	Six sessions over four months	Home	Not stated, but problem solving, positive reinforcement & social support described.	Intrapersonal Interpersonal
Fossum *et al.* 2004 [58]	Counselling for effects of SHSe, monitoring SHSe, changing smoking habits, supporting non-smoking	Child health nurses	Face to face	Not explained	Child health clinic	Self-efficacy	Interpersonal
Zakarian *et al.* 2004 [51]	Behavioural counselling including contracting to reduce SHSe, problem solving, goal setting and self-monitoring	Health educators Nurses Medical assistants	Face to face	Seven counselling sessions over 6 months	Clinic (× 3) Home via phone (× 4)	SLT (Bandura 1977) and behavioural ecological model (Hovell, Wahlgreen & Gehrman, 2002 [ref])	Interpersonal
Emmons *et al.* 2001 [52]	Choice, personal responsibility for change, sel-efficacy, feedback on CO level. Tailored to interest in quitting smoking or reducing SHSe	Health educator	Face to face Phone	One 30–45 motivational interview + four follow up phone calls	Home	MI	Interpersonal
**Anti-smoking socialisation**																			
Kitzman *et al.* 2010 [61]	Nurse Family Partnership. Home visiting program during first two years of child’s life (health promotion, parenting support, developmental screening, planning for pregnancies, education and employment)	Nurse	Face to face	Mean visits during pregnancy = 7 (range 0–118). Mean visits during first two years = 26 visits (range 0–71)	Home	Family partnership model	Intrapersonal Interpersonal

### 3.2. Environmental Tobacco Smoke (ETS) Interventions

Twelve articles reporting on ten studies of family based interventions to reduce ETS were located (Table 3). The majority of studies focused on SHSe reduction, and used an RCT design. Participant retention ranged from 76% to 88%.

#### 3.2.1. Target Populations

The studies targeted families of young children (1–5 years) or those pregnant or caring for infants [49,50,51,52]. Four studies targeted populations with lower socioeconomic status [51,52,53,54] and one study targeted parents of infants at high risk for asthma [55,56,57]. The numbers of participants ranged from 41 to 545.

#### 3.2.2. Interventions

Specific details of the intervention content were not always well described (Table 4). One program used a previously validated SHSe intervention program [53]. The remaining studies developed new interventions or materials using a range of strategies to engage with families such as motivational interviewing [50,52,53,54] or counselling [49,51,55,56,57,58,59]. Four studies used some form of biochemical monitoring and feedback as part of the intervention including home air quality [50,52,54] and child urine cotinine [49].

The studies provided limited information regarding personnel responsible for implementation of the intervention. Most studies reported use of research staff for the intervention, but few provided additional details of professional background. Methods of communication included a mixture of face to face counselling or education, supplemented with telephone support and written materials.

There was considerable variation in intensity of interventions ranging from a single prenatal visit [55,56] to seven counselling sessions over a 6 month period [51]. Little information on session length was provided. The majority of interventions took place, either partially or wholly, in participants’ homes.

The conceptual framework underpinning interventions was not consistently described. Motivational interviewing, the transtheoretical model of behaviour change, social learning theory and the behavioural ecological model were named.

#### 3.2.3. Outcomes

Eight studies used biochemical measures either as a primary outcome for the study, or as a secondary outcome to validate parental self-report of smoking behaviour, including household and child measures (Table 3). Biochemical measures based in the household included air particulate matter (PM_2.5_) [54] and household nicotine levels [52], while child biochemical measures included urine cotinine [49,59], hair nicotine concentration [50,59] and salivary cotinine [54]. One study used maternal salivary cotinine as a secondary outcome measure to verify maternal self-report outcomes [58].

Parent self-report of smoking behaviour was frequently included as an outcome measure, but the assessment varied considerably. One study asked parents to estimate the number of maternal cigarettes that the child was exposed to in one week [51], while another study sought parent reports of the number of household cigarettes that a child was exposed to in one month [60]. Other approaches included parent estimate of the frequency of SHSe avoidance [61], the introduction of household smoking bans [49] or child SHSe exposure before and after birth [55,56,57]. Four studies included current parent current smoking or cessation status [51,52,55,56,57,58]. Two studies included an assessment of maternal knowledge of SHSe and smoking risk [50,60],

#### 3.2.4. Effectiveness

Most studies reported positive results following interventions. These included increased self-reported household restrictions on smoking, decreased cigarette consumption, or avoidance of SHSe [49,51,53,60]. Some confirmation was validated through decreased cotinine levels [52,58,59] or improved air quality [54]. There were no significant changes in parent report of smoking cessation in these studies.

### 3.3. Anti-Smoking Socialisation Interventions

One study analysed the impact of a family-based intervention on children’s smoking behaviour later in life [61] (Table 3 and Table 4). This longitudinal RCT investigated the effect of a two year home visiting model (Nurse Family Partnership) during pregnancy and infancy (through age 2) on the use of substances by children at age 12 years. The Nurse Family Partnership model uses an individualised family approach to improving the outcomes of pregnancy through health promotion of maternal health behaviours, promoting effective parental care and enhancing parent outcomes in pregnancy planning, education and finding employment. While no specific data on tobacco use was described, outcome measures included first born child self-report of substances use at 12 years of age. Children of mothers participating in Nurse Family Partnership were less likely to have used substances, to have used fewer of these substances and to have used these substances for fewer days.

## 4. Discussion

Family based interventions for smoking cessation, relapse prevention and ETS reduction have taken place in a wide range of contexts, targeting families at different stages of family life. Heterogeneity among approaches to interventions, target populations, contexts and efficacy makes it difficult to draw firm conclusions about the best approach. However, interventions for parent smoking cessation and relapse prevention seem to have been less successful than interventions to reduce SHSe. No studies were found that considered third hand smoke contamination.

Whilst it is tempting to argue that SHSe reduction interventions should be considered as an element of any family based intervention, there is some evidence that interventions that try to address more than one element of a smoke free home or are based on universal precautions for substance abuse may be less effective than those that focus on a single target [28]. In previous reviews, both Patnode *et al.* [25] and Rosen *et al.* [62] observed that smoking cessation interventions were more likely to be effective when the focus was on smoking cessation only. At the same time, it is important to recognise that smoking cessation is difficult to achieve and commonly requires multiple quit attempts [63]. In the meantime, ETS reduction remains an important harm reduction strategy.

For studies that targeted parents in pregnancy and early parenthood, the focus was more likely to be on maternal smoking, due to the higher risks from prenatal and postnatal exposure. Early pregnancy and transition to parenting are often perceived to be a powerful motivator for change in health behaviour, but this may be counter-balanced by demographic factors in the smoking trajectory of women during their childbearing and childrearing years related to maternal age, education, ethnicity and socioeconomic status [64,65]. Smoking is often generational and embedded in social network [66]. The smoking of fathers and other family members should not be overlooked. For example, fathers are increasingly taking on primary care roles, and the transition to becoming a parent may also be a motivator to change smoking behaviour [67].

There is some indication that parents of infants or very young children may not be as responsive to intervention as parents of children in the pre-school to school age range [68]. Parents of infants are making their first transition to parenting or coping with the new infant in the context of an already busy family life. Nonetheless, they should not be excluded from interventions as they indicate that they are receptive to the message, and can increase knowledge, even though they may not be ready to implement change [40]. More programs that compare interventions with families at different stages of development (e.g., pregnancy/first year and children over 1 year) are required.

Surprisingly few studies seem to have explicitly considered any of the parenting or family based theories in the development and delivery of their interventions. The positive results reported by Phillips *et al.* [35] suggest that including such theoretical frameworks may be useful in increasing parent motivation for change when used in conjunction with other smoking behaviour interventions in the pre and postnatal period. Furthermore, the interventions used individual techniques, such as motivational interviewing or counselling. This is unsurprising, as few studies truly considered the wider family as part of their target group, yet intrapersonal factors such as knowledge, attitudes, beliefs and values are affected by relationships with others [69].

Interventions that are “family based” should incorporate or offer both intra- and interpersonal level interventions and need further consideration in the context of family based interventions. Given that social cohesion and support is an important factor in continuing abstinence, [70], the importance of interventions that are truly inclusive of the family, not just the smoking parent, are required. Reviews of older children and families have reported studies that included a wider community component in their intervention, and there is some evidence that multi-sector programs that encompass individual, family and community contexts may be more likely to succeed [26]. However, the number of studies are limited and conducted mainly in Western developed countries and have yet to assess efficacy in families with younger children. Consideration of extended family and community level interventions may be critical in the development and delivery of interventions in developing countries as these levels of intervention may be more cost-effective and culturally appropriate [71].

Given the decrease in adult smoking in Western developed countries, it would seem appropriate to target families where smoking is more likely, particularly those of lower socioeconomic status. Yet, little is understood about the best ways in which to reach such families [72]. Depending on their circumstances, families with vulnerabilities may need more support that is offered in brief or individual programs [73]. For example, few studies considered increased availability, access to, or financial support for nicotine replacement therapy.

The use of biochemical markers and environmental air monitoring as either an intervention or outcome measure may be contentious. There is considerable cost associated with these methods and some evidence that parent self-report is a reasonably successful alternative when cost limitations prohibit the use them. Furthermore, such methods may not detect small changes in exposure level over time and monitoring of the control group participants may have an intervention effect [62]. In this review, some studies using biochemical markers or environmental monitoring reported higher refusal rates [50] and of parents who did participate, some would not consent or did not complete biochemical monitoring [38,57] or did not complete. While not conclusive, it is possible that some families may not be comfortable with the level of intrusion that biochemical or environmental monitoring might entail. The use of such devices may exacerbate the sense of stigma associated with being a smoker and thus affect participation in research [73]. Studies that explore parental perceptions of biochemical and environmental monitoring as either intervention or outcome are absent from the literature.

### Limitations

Limitations of this review include the English language-only literature inclusion and search terminology that did not encompass substance use or drug references. The majority of studies included in this review were from Western developed countries. More studies are needed from developing countries, particularly as this is a “growth” area for tobacco use. Some studies were excluded because child age data was not provided.

## 5. Conclusions

Smoking cessation interventions are critically important and there is a need for a range of interventions that are both tailored and targeted to specific populations and also opportunistic models of interventions that can be activated during clinical encounters. As in many non-pharmacological interventions, quality of reporting challenges identification of intervention elements. Based on this review, interventions that target the social and psychodynamics of the family should be considered further, particularly with regard to vulnerable populations.
